# Protein *O*-GlcNAcylation in Metabolic Modulation of Skeletal Muscle: A Bright but Long Way to Go

**DOI:** 10.3390/metabo12100888

**Published:** 2022-09-22

**Authors:** Yang Liu, Yajie Hu, Shize Li

**Affiliations:** College of Animal Science and Veterinary Medicine, Heilongjiang Bayi Agricultural University, Daqing 163319, China

**Keywords:** *O*-GlcNAcylation, skeletal muscle, hexosamine biosynthetic pathway, metabolism, exercise

## Abstract

*O*-GlcNAcylation is an atypical, dynamic and reversible *O*-glycosylation that is critical and abundant in metazoan. *O*-GlcNAcylation coordinates and receives various signaling inputs such as nutrients and stresses, thus spatiotemporally regulating the activity, stability, localization and interaction of target proteins to participate in cellular physiological functions. Our review discusses in depth the involvement of *O*-GlcNAcylation in the precise regulation of skeletal muscle metabolism, such as glucose homeostasis, insulin sensitivity, tricarboxylic acid cycle and mitochondrial biogenesis. The complex interaction and precise modulation of *O*-GlcNAcylation in these nutritional pathways of skeletal muscle also provide emerging mechanical information on how nutrients affect health, exercise and disease. Meanwhile, we explored the potential role of *O*-GlcNAcylation in skeletal muscle pathology and focused on its benefits in maintaining proteostasis under atrophy. In general, these understandings of *O*-GlcNAcylation are conducive to providing new insights into skeletal muscle (patho) physiology.

## 1. Introduction

**Figure 1:**
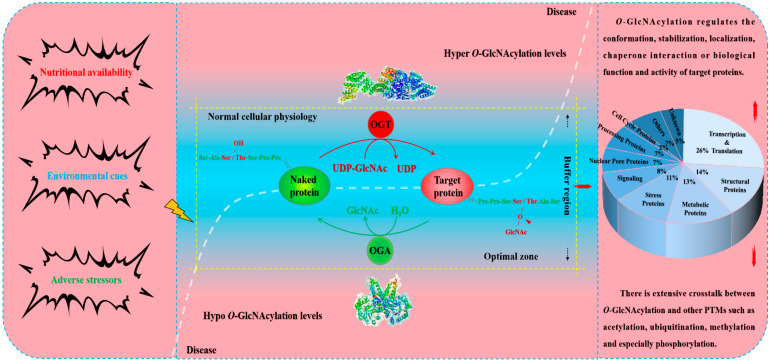
Protein *O*-GlcNAcylation homeostasis is maintained by its only pair of process enzymes, OGA and OGT. To date, thousands of proteins have been identified as *O*-GlcNAcylated, and these proteins are diverse including enzymes, transcription factors, signaling proteins, mitochondrial proteins, among others. *O*-GlcNAcylation homeostasis of these proteins is precisely and rapidly regulated only by OGT and OGA. Faced with mild stress and acute adverse stimulation, OGT and OGA form a “buffer” to make *O*-GlcNAcylation fluctuate adaptively within the “optimal zone”. Strong stress or chronic adverse stimulation beyond the buffer threshold of OGT and OGA results in *O*-GlcNAcylation levels exceeding the optimal zone. Both hyper- and hypo-*O*-GlcNAcylation beyond the “buffer region” lead to cellular dysfunction and other harmful effects.

Various post-translational modifications (PTMs) continuously and dynamically amplify the proteome to produce a wide variety of protein forms and their biological functions, thereby harmonizing the proteome with the needs of the organism [1,2,3]. Glycosylation is one of the most widespread and abundant PTMs in living organisms, encompassing different types involving complex metabolic networks [4,5]. *O*-GlcNAcylation is a dynamic, reversible and atypical *O*-glycosylation that involves only the binding of a single *N*-acetylglucosamine (GlcNAc) to the serine and threonine residues of the target protein via a *β*-configuration *O*-glycosidic bond [6]. *O*-GlcNAc transferase (OGT) and *O*-GlcNAcase (OGA) are the only pair of mutually antagonistic process enzymes for protein *O*-GlcNAcylation [7]. OGT transfers GlcNAc from UDP-*N*-acetylglucosamine (UDP-GlcNAc) to the hydroxyl groups in the threonine and serine residues of the target proteins [8]. In contrast, OGA hydrolyzes GlcNAc from the *O*-GlcNAcylated proteins [9]. See Figure 1 for a schematic diagram of the *O*-GlcNAcylation process. Coordination of OGT and OGA accurately and rapidly modulate the *O*-GlcNAcylation cycle of thousands of proteins [10]. It is the biological properties of OGT and OGA that allow the rapid addition and removal of reversible GlcNAc multiple times during the life cycle of the target proteins that confer the high kinetics of *O*-GlcNAcylation [11,12]. UDP-GlcNAc is the only donor substrate providing the required GlcNAc for *O*-GlcNAcylation [13]. UDP-GlcNAc is produced from the hexosamine biosynthetic pathway (HBP) controlled by the input of glucose, glutamine, acetyl-CoA and uridine triphosphate (UTP), making it a sensor molecule for fluctuations in these macromolecules [14]. It is the physiological property of UDP-GlcNAc that endows *O*-GlcNAcylation with a unique high sensitivity to nutrient availability and thus serves as a critical nutrient sensor for the metabolism of carbohydrate, amino acid, lipid and nucleotide [15]. Meanwhile, *O*-GlcNAcylation senses external environmental disturbance and internal adverse stimulation, acts as a stress receptor integrating signaling pathway inputs from different partners, and generates spatiotemporal-specific adaptive molecules and physiological responses by targeting multiple substrates [16,17].

*O*-GlcNAcylation exists in almost all living organisms, and it is distributed in almost all human tissues, even in saliva and urine [18]. *O*-GlcNAcylation occurs in almost all cellular compartments, and its first discovery in 1984 overturned the conventional knowledge that glycosylation occurs only in the endoplasmic reticulum and Golgi apparatus [19]. It also shows that almost all functions of proteins in regulating various cellular processes are covered. This importance is reflected again by the of OGA and OGA defects lethality [20,21]. *O*-GlcNAcylation affects the function, activity, stabilization, localization and chaperone interactions of target proteins, thereby participating in the regulation of a series of cellular biological processes such as immunity [22,23], inflammation [24], autophagy [25,26], apoptosis [27], stemness [28,29], transcription [30], translation [31], signal transduction [32], mitochondrial function [33], epigenetics [10,34], chromatin remodeling [35], metabolic reprogramming [36] and cellular stress responses [37,38]. Meanwhile, *O*-GlcNAcylation has surprisingly extensive crosstalk with other PTMs such as phosphorylation, acetylation, ubiquitination, methylation, and so on [39]. In particular, the abundance and cycle time scales of *O*-GlcNAcylation are very similar to those of phosphorylation and thus form a yin–yang relationship that may be co-occurring or competing and negatively or positively affect each other through their interaction [40,41]. In addition, *O*-GlcNAcylation homeostasis is essential for the maintenance of the normal physiological function of cells, tissues and organs. Abnormal *O*-GlcNAcylation levels are closely associated with the pathogenesis and progression of various diseases such as neurodegenerative diseases [42], cardiovascular diseases [43,44], aging [45], obesity [46,47], amyotrophy [48], cancers [49], diabetes and diabetic complications [50].

To date, thousands of *O*-GlcNAcylated proteins have been identified in skeletal muscle, including contractile proteins, structural proteins, cytoskeletal proteins and sarcolemma protein as well as metabolic enzymes, transcription factors and signaling proteins and mitochondrial proteins [51,52,53,54,55]. This suggests a unique and central physiological role for *O*-GlcNAcylation in skeletal muscle. Recent data also suggest that *O*-GlcNAcylation is involved in different cellular processes in skeletal muscle such as regulation of contractile and structural properties, maintenance of energy metabolic homeostasis, and mediation of insulin resistance [48,56,57]. Meanwhile, *O*-GlcNAcylation plays a potential role in many diseases related to skeletal muscle defects such as neuromuscular diseases and amyotrophy [58,59]. What we are interested in is that fast and slow skeletal muscles have different fine characteristics of *O*-GlcNAcylation during rest, exercise or muscle atrophy [60]. However, these effects and features of *O*-GlcNAcylation are neglected or underestimated. This review highlights the spatiotemporal regulation of *O*-GlcNAcylation and promotes metabolic flexibility in skeletal muscle to maintain energy metabolic homeostasis. Meanwhile, this review emphasizes some consensus on the effects of exercise frequency, intensity, type and duration on *O*-GlcNAcylation and its process enzymes. In addition, this review discusses the potential role of *O*-GlcNAcylation in skeletal muscle-related disorders. These understandings of *O*-GlcNAcylation contribute to provide emerging insights into the (patho)physiology of skeletal muscle.

## 2. *O*-GlcNAcylation Is an Essential Metabolic Modulator in Skeletal Muscle Physiology

Skeletal muscle fibers are very abundant and metabolically active, particularly during exercise [61]. Skeletal muscle accounts for approximately 40% of the total body weight of mammals and contains up to 75% of total body proteins and 30% of the resting metabolic rate in adults [62]. Skeletal muscle is the main contributor of metabolic changes caused by exercise. Maximum exercise increases the whole-body metabolic rate 20 times higher than the resting value, while the adenosine triphosphate (ATP) conversion rate in working skeletal muscle is more than 100 times higher than that at rest [63]. The metabolic flexibility of skeletal muscle allows it to increase energy supply and provide sufficient “fuel” for contraction [64,65]. The importance of carbohydrate as a fuel source in exercise has been known, and the main source of these carbohydrate is glycogen reserve of skeletal muscle during exercise [66,67]. In addition, skeletal muscle actively secretes myogenic protein to exert autocrine, paracrine or endocrine functions to convey its energy demand to other organs, including adipose tissue, the liver, pancreas, cardiovascular system, brain, bone and skin, thus causing crosstalk [68,69]. *O*-GlcNAcylation plays a decisive role in skeletal muscle glucose homeostasis as a cellular trophic sensor by participating in glycolysis [70,71], tricarboxylic acid (TCA) cycle [36], insulin signaling [72,73] and glycogen metabolism [74,75].

### 2.1. O-GlcNAcylation Is an Extremely Sensitive Sensor for the Nutrition Availability in Skeletal Muscle via the HBP

**Figure 2:**
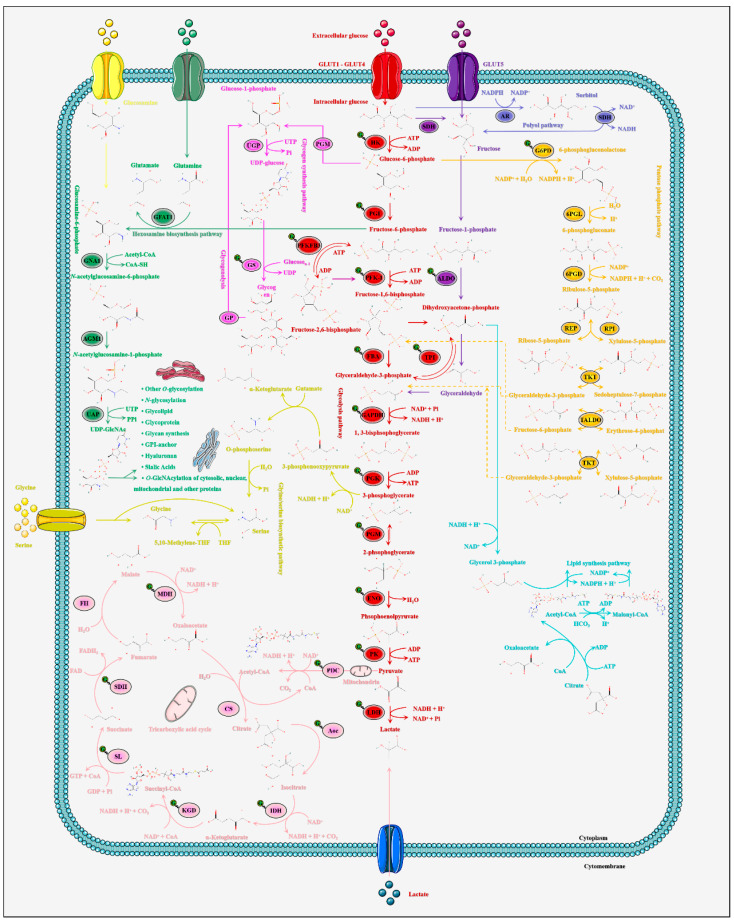
*O*-GlcNAcylation is linked by the HBP to nutrient availability and thus its regulation of the complex metabolic network. Two percent to 3% of intracellular glucose enters the HBP and undergoes a series of chemical reactions to produce the end product UDP-GlcNAc. The UDP-GlcNAc in the nucleus, cytoplasm and membrane is utilized for *O*-GlcNAcylation. UDP-GlcNAc makes the dynamic *O*-GlcNAcylation extremely sensitive to changes in cellular nutrients used as a sensor of the functional state of multiple pathways such as glycolysis, TCA cycle, pentose phosphate pathway (PPP), glycogen synthesis and catabolism, etc. Meanwhile, the interconversion of intermediates between the HBP, polyol pathway, fructose, PPP, glycogen, glycolysis and TCA cycle greatly enhanced the nutritional sensitivity of *O*-GlcNAcylation. A portion of UDP-GlcNAc is transported to the endoplasmic reticulum and the Golgi apparatus while being used for other *O*-glycosylation and *N*-glycosylation. Other fractions of UDP-GlcNAc are used for the biosynthesis of proteoglycans, hyaluronic acid, glycolipids, glycolphosphatidylinositol anchoring, etc.

It has been a popular view for many years that the cellular *O*-GlcNAcylation cycle is strictly controlled by HBP flow and UDP-GlcNAc concentration [76]. The HBP is a conventional branch of the glucose metabolic pathway and its flow varies with extracellular glucose level. See Figure 2 for the detailed process of HBP [77]. Initially, extracellular glucose is transported into the intracellular through glucose transporter 1–4 (GLUT1-4) [78]. Therefore, the *O*-GlcNAcylation cycle is strictly controlled by the flow of glucose through the HBP. Subsequently, intracellular glucose was phosphorylated to glucose-6-phosphate by hexokinase, and then glucose-6-phosphate was further isomerized to fructose-6-phosphate by phosphoglucose isomerase [79]. Three to 5% of fructose-6-phosphate was added with an amino group from a glutamine to synthesize glucosamine-6-phosphate and a glutamate by glutamine fructose-6-phosphate amidotransferase-1 [80]. This enzymatic reaction is the rate-limiting step of HBP, and glutamine fructose-6-phosphate amidotransferase-1 is also the critical rate-limiting enzyme of HBP. Glutamine is necessary for this enzymatic reaction, but this restriction is bypassed by glucosamine as an extended supplement [81]. Glucosamine-6-phosphate acetyltransferase acetylates glucosamine-6-phosphate to *N*-acetylglucosamine-6-phosphate using acetyl-CoA [82]. Then, *N*-acetylglucosamine-6-phosphate is catalytically translocated to *N*-acetylglucosamine-1-phosphate by *N*-acetylglucosamine-phosphate mutase 1. Finally, UTP is then utilized by UDP-*N*-acetylglucosamine pyrophosphorylase-1 to convert *N*-acetylglucosamine-1-phosphate into UDP-GlcNAc and release Pi [83]. UDP-GlcNAc, as the end product of HBP, involves the participation of glucose, glutamine, uridine, acetyl-CoA and ATP [84]. Therefore, UDP-GlcNAc is sensitive to the fluctuation of all these nutrients as a “sensor molecule” [85]. The GlcNAc provided by UDP-GlcNAc is necessary and irreplaceable for *O*-GlcNAcylation. It is this physiological property that makes *O*-GlcNAcylation a major metabolic node that integrates the metabolism of carbohydrates, amino acids, lipids and nucleotides [86]. On this basis, combined with the high dynamic nature of *O*-GlcNAcylation, it makes it a highly sensitive and rapidly responsive metabolic sensor of nutrient availability reporting multiple pathway functional states [87]. Overall, HBP and UDP-GlcNAc link altered metabolisms and *O*-GlcNAcylation, providing an important mechanism for cells to perceive and respond to nutritional availability.

### 2.2. O-GlcNAcylation Is the Pivotal Modulator of Glucose Metabolic Homeostasis in Skeletal Muscle

During low-to-moderate-intensity exercise, the main fuel sources supplying skeletal muscle are glucose and are heavily influenced by insulin and glucose utilization increases progressively with increasing exercise intensity until near maximum intensity [64]. Several recent studies have demonstrated that *O*-GlcNAcylation controls early glucose metabolism in skeletal muscle [48,72,88,89]. The fuel metabolism and energy homeostasis of skeletal muscle are greatly dependent on the glucose uptake and disposal. *O*-GlcNAcylation alters GLUT4 translocation, prevents GLUT4 phosphorylation, controls GLUT4 downstream signal transduction, or directly controls vesicle proteins to sustain glucose absorption [90,91]. Meanwhile, *O*-GlcNAcylation of hypoxia-inducible factor-1 enhances GLUT1 transcription and thereby regulates glucose uptake [92]. Additionally, *O*-GlcNAcylated hexokinase IV or glucokinase upregulates their expression, which is of positive significance in controlling glucose flow [93]. Similar alterations have taken place in *O*-GlcNAcylated phosphoglucose isomerase [88]. *O*-GlcNAcylation of glucose-6-phosphate dehydrogenase at Ser^84^ enhances its catalytic activity, thereby forcing a shift in glucose flow to the pentose phosphate pathway [94]. The *O*-GlcNAcylation of phosphofructokinase 1 at Ser^529^ inhibits its activity and redirects glucose flow into the pentose phosphate pathway, which also reduces glycolytic glucose flow [71]. The *O*-GlcNAcylation 6-phosphofructo-2-kinase/fructose-2,6-bisphosphatase at Ser^172^ competes with its phosphorylation to modulate glycolysis [95]. In fact, almost all enzymes involved in the glycolysis pathway are *O*-GlcNAcylated such as fructose-1,6-bisphosphate aldolase and glyceraldehyde-3-phosphate dehydrogenase [7]. Transcription of these enzymes is also mediated by *O*-GlcNAcylation of Sp1 [96]. Another critical metabolic enzyme in the glycolytic pathway, phosphoglycerate kinase 1, is also *O*-GlcNAcylated at Thr^255^, which enhances its activity to promote glycolysis and translocation to mitochondria to inhibit the TCA cycle [36]. The downstream phosphoglycerate mutase and α-enolase were also identified as *O*-GlcNAcylated, but their functions need to be further investigated. The *O*-GlcNAcylation of pyruvate kinase M2 at Thr^405^ and Ser^406^ destroys its stability, reduces its activity and causes its nuclear translocation [70]. These findings imply that *O*-GlcNAcylation regulates the skeletal muscle’s glycolysis process fundamentally [97]. In addition, the terminal lactate dehydrogenase is also *O*-GlcNAcylated, indicating that *O*-GlcNAcylation modulates the utilization of glycolysis end products [88]. Skeletal muscle is the largest glycogen reservoir and has four times the capacity of the liver [98]. Glycogen synthesis and catabolism contribute to the maintenance of energy homeostasis in skeletal muscle, and *O*-GlcNAcylation is a regulator of glycogen metabolism. For example, the *O*-GlcNAcylation of glycogen synthase kinase 3β (GSK3β) competes with its phosphorylation at Ser^9^ to inhibit its activity [75,99]. Similar regulation also occurs on the *O*-GlcNAcylated glycogen synthase (GS) [100]. In addition, OGT indirectly regulates the global metabolism of skeletal muscle through mediated expression of interleukin-6 and interleukin-15 [57,101]. Skeletal muscle is also one of the main sites of energy-producing lipid metabolism. Free fatty acids released by lipolysis in the liver and adipose tissue have a significant contribution to the substrate supply of muscle contraction during exercise. The increased HBP flux and *O*-GlcNAcylation levels are associated with greater fatty acid oxidation in the heart, possibly through *O*-GlcNAcylation of CD36 (the fatty acid transporter) [102]. Similar results were seen in adipocytes and short OGA, a splice variant of OGA, is associated with lipid droplets. In addition, fatty acids and their derivatives may play different regulatory roles on the quality and function of skeletal muscle [61,65]. The *O*-GlcNAcylation of carbohydrate-responsive element-binding protein, farnesoid X receptor, sterol regulatory element binding protein 1, perilipin 1, liver X receptor-α, fatty acid synthase and other proteins is also involved in the regulation of lipid metabolism, but this information comes from the liver and adipose tissue, etc., rather than skeletal muscle [46,103,104,105,106,107]. However, the connection of information and energy between skeletal muscle and other organs or tissues is close and frequent [69]. Therefore, this information is of certain value for understanding the modulation of *O*-GlcNAcylation on skeletal muscle energy homeostasis, but we do not discuss it further.

### 2.3. O-GlcNAcylation Is the Precise Spatiotemporal Regulator of Insulin Signal Transduction in Skeletal Muscle

**Figure 3:**
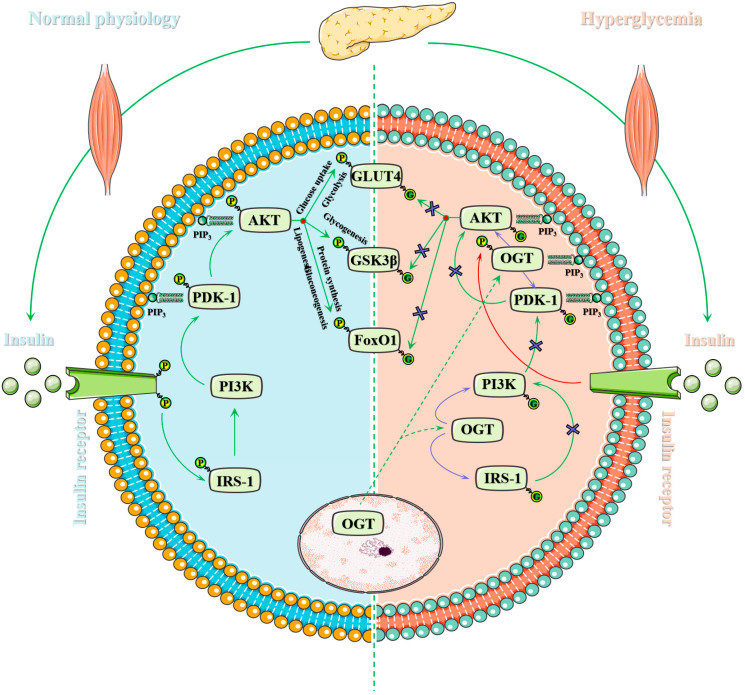
Spatiotemporal regulation of *O*-GlcNAcylation on insulin signal transduction of skeletal muscle. Skeletal muscle is the major insulin-sensitive tissue, responsible for about 75% of insulin-stimulated glucose uptake in the whole body, which is critical for glucose homeostasis and energy metabolism. The harmonious balance between *O*-GlcNAcylation and phosphorylation is necessary for the normal operation of insulin signaling under normal physiology, and the disruption of this homeostasis leads to the impairment of insulin-signaling cascade. *O*-GlcNAcylation has been described as protecting cells from acute stress but harmful to chronic and persistent stress in various organs or tissues. For example, almost all insulin-sensitive tissues have a hyper *O*-GlcNAcylation level and many related complications are under hyperglycemia. This adverse effect is achieved through impaired glucose utilization or glucose toxicity paradigms and may lead to the progression of a variety of diseases. Therefore, chronic elevation of *O*-GlcNAcylation is considered as a mechanism for the development of insulin resistance.

Skeletal muscle is the main tissue for glucose treatment under insulin-stimulated conditions and thus plays a pivotal role in blood glucose control and systemic metabolic homeostasis [108]. It has long been generally recognized that there is a strong correlation between the excessive HBP flow and UDP-GlcNAc level and skeletal muscle insulin resistance and diabetic pathology [109,110,111]. The concentration of UDP-GlcNAc and HBP flow have a significant impact on OGT’s catalytic activity [112]. Meanwhile, co-infusion of insulin and glucosamine resulted in increased HBP flux and UDP-GlcNAc levels enhancing overall *O*-GlcNAcylation levels and many *O*-GlcNAcylated proteins on skeletal muscle [113,114]. Interestingly, almost all of the major effector molecules of the insulin signaling pathway are *O*-GlcNAcylated, such as insulin receptor substrates-1 (IRS-1), phosphoinositide 3-kinase (PI3K), 3-phosphoinositide-dependent kinase-1 (PDK-1), protein kinase B (AKT), and forkhead box O1 (FoxO1). Therefore, it is self-evident that this chronically elevated *O*-GlcNAcylation disrupts the insulin signal transduction in skeletal muscle [115]. That is, the mechanism of HBP-induced skeletal muscle insulin resistance is that the increased *O*-GlcNAcylation of participants in insulin signaling disrupts the balance of its antagonism with phosphorylation on these proteins to negatively regulate insulin signaling [40,116]. See Figure 3 for more details. Insulin receptor binds to insulin to make itself phosphorylated on the cytomembrane under normal physiological conditions, which cause subsequent signal cascade [117]. Subsequently, IRS-1 is phosphorylated, resulting in PI3K activation [118]. Then, activated PI3K produces phosphatidylinositol-3,4,5-trisphosphate (PIP_3_) on the cytomembrane, which binds to AKT and PDK1 [119,120]. AKT is phosphorylated at the Thr^308^ by PDK1 to activate [121]. Downstream target proteins are phosphorylated by activated AKT for activation or inhibition [122]. Phosphorylation inhibition of downstream GSK3β and GS promotes glycogen synthesis [74]. FoxO1 phosphorylation inhibits gluconeogenesis gene transcription, thereby reducing gluconeogenesis [123]. Membrane translocation of GLUT4 is promoted to glycolysis and glucose uptake [124]. Ultimately, these pathways make the elevated glucose return to homeostasis [125]. However, PIP_3_ also recruits OGT to the cytomembrane through the strong interaction with the phosphoinositide-binding domain of OGT under diabetes or another insulin-insensitive state such as the prolonged postprandial response [126]. Meanwhile, OGT is phosphorylated and enhances its activity through IR [127]. Subsequently, OGT catalyzes the dynamic *O*-GlcNAcylation of IRS, PDK1, AKT, FoxO1 and other insulin signal molecules [128,129]. The *O*-GlcNAcylation of IRS-1 at Ser^1101^ and IRS-2 at Ser^1149^ inhibits their phosphorylation at the same site, resulting in the attenuation of insulin signal [128]. The *O*-GlcNAcylation of AKT at the Thr^305^ and Thr^312^ inhibits its phosphorylation at Thr^308^ through disrupting the interaction between AKT and PDK1 [130]. The *O*-GlcNAcylations of PI3K and PDK1 are also implicated in insulin-signaling attenuation. In fact, the precise control of insulin-signaling players by dynamic antagonism of phosphorylation and *O*-GlcNAcylation goes far beyond this. For example, *O*-GlcNAcylation of protein-tyrosine phosphatase 1B regulates its phosphatase activity and affects insulin signaling, thus causing insulin resistance [131,132]. *O*-GlcNAcylation of S6 kinase beta1 inhibits its phosphorylation and mTORC1 signaling, thereby causing insulin resistance [133]. In addition, *O*-GlcNAcylation of cAMP-response element binding protein (CREB) crosstalk with its phosphorylation [134,135]. *O*-GlcNAcylation of CREB-regulated transcription coactivator 2 at Ser^70^ and Ser^171^ inhibits the phosphorylation of these two sites, thereby facilitating their nuclear translocation and binding to CREB [136].

### 2.4. O-GlcNAcylation Is the Pivotal Maintainer of the TCA Cycle and Mitochondrial Homeostasis in Skeletal Muscle

Skeletal muscle is rich in mitochondria and relies heavily on oxidative phosphorylation to generate energy [137]. During vigorous exercise, intramuscular oxygen consumption and local blood flow increased significantly by more than 30-fold and the estimated TCA cycle flux increased by 70- to 100-fold [138]. Many studies have shown that changes in cellular *O*-GlcNAcylation levels alter mitochondrial function, especially under stress [139,140]. Mitochondrial OGT, one of the OGT splice variants, has a unique mitochondrial targeting sequence [141]. Although these data have shown that mitochondrial proteins are targets of *O*-GlcNAcylation, *O*-GlcNAcylation studies were initially hampered by the unclear mechanism of UDP-GlcNAc translocation to mitochondria. It was once thought that OGT in mitochondria was rare or even non-existent. This obstacle was overcome by Gerald W. Hart in 2015, whose study found that the mitochondrial pyrimidine nucleotide carrier 1 is responsible for efficient transport of radioactive ^3^ H UDP-GlcNAc into mitochondria [45]. This study also demonstrates the presence of mitochondria-specific OGA, which also proves the existence of an active *O*-GlcNAcylation cycle. This *O*-GlcNAcylation biology’s founder has once again advanced the understanding of *O*-GlcNAcylation in mitochondria. Subsequently, *O*-GlcNAcylation on many mitochondrial respiratory chain complexes was identified such as complex I, II, and IV, which led to a decrease in their activity and mitochondrial calcium and cellular ATP content [142]. Meanwhile, eleven *O*-GlcNAcylated proteins were identified in mitochondria, all of which are metabolic enzymes involved in the TCA cycle and ATP synthesis [143]. It should be emphasized that almost all metabolic enzymes involved in the TCA cycle on mitochondrion are also *O*-GlcNAcylated such as pyruvate dehydrogenase, aconitase, isocitrate dehydrogenase, ɑ-ketoglutarate dehydrogenase, succinyl-CoA ligase, succinate dehydrogenase and malate dehydrogenase, except for citrate synthase and fumarase [88,144]. Further, two proteomic studies identified up to hundreds of mitochondrial proteins as targets of *O*-GlcNAcylation, most of which are involved in a variety of biological processes such as oxidative phosphorylation, urea cycle, fatty acid oxidation and calcium regulation [145,146,147]. These data emphasize the importance of *O*-GlcNAcylation as a regulatory molecule of mitochondrial metabolism and respiration. Meanwhile, *O*-GlcNAcylation modifies a variety of mitochondrial proteins to change the motility, morphology and quality of mitochondria [148]. For example, *O*-GlcNAcylation of peroxisome proliferators-activated receptor γ coactivator ɑ is beneficial to maintain mitochondrial biogenesis [149]. *O*-GlcNAcylation of Milton in response to the glucose availability mediates changes in neuronal mitochondrial mobility [150]. Meanwhile, *O*-GlcNAcylation of dynamin-related protein 1 at Thr^585^ and Thr^586^ inhibits its phosphorylation at Ser^637^, which promoted its translocation to mitochondria and increased fragmentation as well as decreased membrane potential [151,152,153]. These data have demonstrated that *O*-GlcNAcylation is closely related to mitochondrial function. On the other hand, *O*-GlcNAcylation should play an important role in mitochondrial oxidative stress. For example, Increased *O*-GlcNAcylation levels mediated by glucosamine treatment and OGT overexpression attenuated hydrogen peroxide-induced mitochondrial membrane potential and enhanced the recruitment of the anti-apoptotic protein B-cell lymphoma-2 (Bcl-2) to mitochondria [154]. In addition, a subsequent study demonstrated that Bcl-2 is *O*-GlcNAcylated [155]. *O*-GlcNAcylation of 8-oxoguanine DNA glycosylase impairs mitochondrial DNA damage repair under oxidative stress [156]. *O*-GlcNAcylation of voltage-dependent anion channel 1 attenuates oxidative stress-induced loss of mitochondrial membrane potential [157]. *O*-GlcNAcylation of superoxide dismutase 2 has a similar effect [158].

## 3. The Fine Characteristics of *O*-GlcNAcylation, Skeletal Muscle Fibre Types and Exercises

Skeletal muscle is a heterogeneous cell population consisting of different types of muscle fibers that is classified as slow-twitch muscle fibers (type I) or fast-twitch muscle fibers (types IIa, IIx, and IIb) according to the contractile property of “time-to-peak tension” or “twitch” characteristics [159]. The heterogeneity allows the same muscle to be used for a variety of tasks, from continuous low-intensity activities (such as postures), to repetitive submaximal contractions (such as exercise), to fast and intense maximal contractions (such as jump, kick) [160]. Each muscle fiber type has its own specific and unique contribution to respond to functional needs in the best way [161]. Type I is heavily dependent on oxidative metabolism for the continuous work, and Type I has a red appearance due to its rich myoglobin and oxidizing enzymes [162]. Type II is characterized by glycolytic metabolism for the phased work, but types IIa, IIx and IIb have different degrees of oxidation and glycolytic capacity. This also leads to the intermediate appearance of type IIa and the white appearance of type IIx and IIb [163]. Meanwhile, type IIb is mainly exist in rodents, and types IIa and IIx are classified in humans [164]. It is the metabolic and morphological phenotype of each skeletal muscle fiber type coupled with the essential regulation of *O*-GlcNAcylation on skeletal muscle metabolic homeostasis and structural and contractile properties leading to the unique fine characteristics of *O*-GlcNAcylation in each type of skeletal muscle fiber [48,72,144,165,166]. In fact, the modulation of *O*-GlcNAcylation, OGT and OGA in each muscle fiber type is completely different, which may be attributed to the patterns of metabolic and molecular responses unique to each skeletal muscle fiber type [167]. For example, the global *O*-GlcNAcylation was much higher in slow-twitch muscle at rest [72,168]. Meanwhile, the *O*-GlcNAcylation process enzyme and glutamine fructose-6-phosphate amidotransferase in soleus were higher than those in the extensor digitorum longus muscle, and so were their activities [168,169]. The *O*-GlcNAcylation pattern of each muscle fiber type is also differentiated in health and in diseases such as atrophy and aging [170,171,172]. For example, the overall level of *O*-GlcNAcylation in soleus was decreased under the disuse atrophy model. The activities of OGT and OGA in atrophic soleus muscle increased and decreased respectively at the later stage of this model [169]. Changes of *O*-GlcNAcylation in each muscle fiber type during exercise are more complex and unpredictable. The global *O*-GlcNAcylation in both slow- and fast-twitch muscle levels were unchanged during a single acute exercise, and its fine regulation was only observed on some specific myofilament proteins such as myosin light chain 2 [168,173]. This fine regulation was reversed after recovery. Confusingly, another study showed that the same acute exercise increased the overall *O*-GlcNAcylation, regardless of slow- or fast-twitch muscle [174]. This confusion is attributed to the differentiation of *O*-GlcNAcylation on the metabolic flexibility and glucose utilization and oxidative stress of skeletal muscle in the short term [64,175,176]. Chronic exercise involves the adaptation and remodeling of skeletal muscle [60]. Due to skeletal muscle plasticity, muscle fibers remodel their structural and functional properties and protein homeostasis in response to the different environmental conditions imposed on them, such as nerve stimulation pattern, load intensity, substrate availability and hormone signal changes [177,178,179,180]. Meanwhile, the changes of mechanical strain, calcium flux, ATP turnover, redox balance, ROS production and oxygen pressure caused by contraction are all related to the activation of signal transduction cascades that regulate skeletal muscle plasticity [181,182]. A series of changes in myosin isoforms, protein turnover, metabolism, mitochondrial function, intracellular signaling or transcription occur during skeletal muscle remodeling [183]. These processes also have the close participation of *O*-GlcNAcylation [184,185,186,187,188]. The muscle fiber type is genetically determined during development, and the adaptive “transformation” of muscle fiber type from one to another is still hotly debated [189]. The regulatory effect of *O*-GlcNAcylation on differentiation and myogenesis have been demonstrated [89,190,191,192]. For example, the global *O*-GlcNAcylation reduced in early myogenesis before myoblast fusion and the inactivation of OGA severely interferes with the expression of myogenin and myogenic regulatory factor 4, which indicate that the terminal differentiation program of skeletal myogenesis is negatively regulated by *O*-GlcNAcylation [193]. Hyper *O*-GlcNAcylation of myocyte-specific enhancer factor 2c at Thr^9^ inhibits its DNA binding affinity to the myogenin promoter to attenuate skeletal muscle differentiation [194]. This negative regulation was also eliminated by hypo *O*-GlcNAcylation of myocyte-specific enhancer factor 2d [195]. Skeletal muscle regeneration and repair can provide functional recovery during chronic exercise [196]. *O*-GlcNAcylation contributes to a self-renewal ability of muscle satellite cells after acute injury and plays a pivotal role in ensuring the health and function of skeletal muscle [196]. Although the changes of *O*-GlcNAcylation still largely depend on the skeletal muscle fiber type and exercise program, there is still a long way to go to reach a consensus. Another awkward situation is that the nodes of *O*-GlcNAcylation associated with exercise have been studied more in cardiac muscle and smooth muscle than in skeletal muscle [60,197]. We do not discuss further here.

## 4. *O*-GlcNAcylation, Autophagy and Skeletal Muscle Pathology

**Figure 4:**
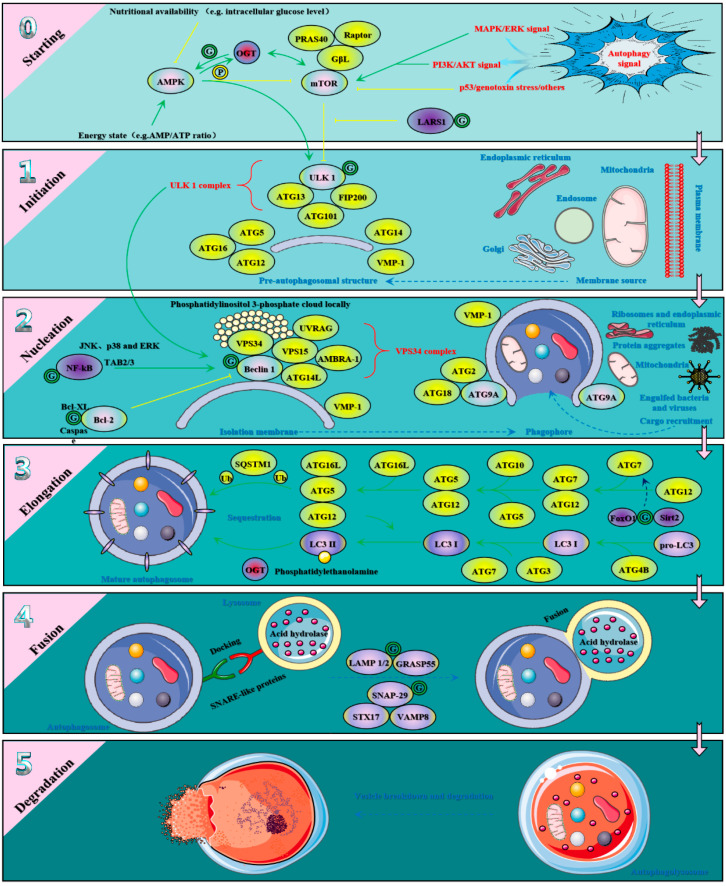
The main target of *O*-GlcNAcylation involved in autophagy process of skeletal muscle. *O*-GlcNAcylation is involved in almost all processes of autophagy including initiation, nucleation, expansion and fusion in addition to degradation. First, there is extremely extensive crosstalk between *O*-GlcNAcylation and autophagic signal transduction. Activation of AMPK in response to energy deprivation inhibits mTOR and activates ULK1. Acute activation of AMPK alters the substrate selectivity and nuclear localization of OGT in myotubes, which is closely related to the phosphorylation of OGT at Thr^444^ by AMPK. In contrast, OGT catalyzes the *O*-GlcNAcylation of α and γ subunits of AMPK, and these modifications increase with increasing AMPK activity. There is crosstalk between *O*-GlcNAcylation and mTOR signaling including their mutual regulation of each other’s stability. Meanwhile, OGT regulates autophagy gene expression and autophagy structure through AKT and FoxO3. At the initiation of autophagy, ULK1-ATG13-ATG101-FIP200 complex and vacuolar protein sorting (VPS34)-Beclin 1 complex coordinate to drive the formation of isolation membrane, which is inhibited by mTORC1 and inactivated by Bcl-2 inactivates, respectively. *O*-GlcNAcylation of ULK1 at Thr^754^ is essential for ATG14L binding and phosphorylation, allowing the activation of VPS34 and leading to the production of a phosphatidylinositol 3-phosphate cloud locally. Beclin-1 and Bcl-2 are targets for *O*-GlcNAcylation. OGT deletion was associated with increased ATG expression and autophagy lysosomes. Inhibition of OGT significantly increased the level of LC3-II and LC3 punctate structure, decreased the level of autophagy substrate SQSTM1, and increased the expression of lysosome-associated membrane protein (LAMP) 1 and 2. These evidences are sufficient to indicate that *O*-GlcNAcylatio plays a pivotal role in regulating the expansion of autophagy. Subsequently, *O*-GlcNAcylation sites of SNAP-29 at Ser^2^, Ser^61^, Thr^130^ and Ser^153^ disturbed the SNAP29-syntaxin-17 (STX17)-vesicle-associated membrane protein 8 (VAMP8) complex, which regulates autophagosome maturation.

There is abundant evidence to support the potential role of *O*-GlcNAcylation in the skeletal muscle pathology [88,198]. For example, the global *O*-GlcNAcylation level in skeletal muscle is increased and its localization is abnormally altered in some human neuromuscular diseases such as muscular dystrophy, myositis and rhabdomyolysis [199]. Abnormal changes of the global *O*-GlcNAcylation level abnormal change under amyotrophic lateral sclerosis, and the progression of this disease is may be attributed to the *O*-GlcNAcylation of the tail domain of neurofilament protein M in spinal cord tissue [200,201]. The *O*-GlcNAcylation of gigaxonin at Ser^272^ and Thr^277^ mediates intermediate filament proteostasis and turnover to regulate human giant axonal neuropathy [202]. UDP-*N*-acetylglucosamine 2-epimerase/*N*-acetylmannosamine kinase (GNE) is a bifunctional enzyme critical for sialic acid biosynthesis [203]. GNE mutations cause GNE myopathy such as hereditary inclusion body myopathy and distal myopathy with rimmed vacuoles, and *O*-GlcNAcylation of GNE at Thr^743^ cooperates with the phosphorylation at the same site to affect its efficiency and activity of GNE [204]. However, the abnormal overall *O*-GlcNAcylation levels is the major pathogenic factors in skeletal muscle atrophy [48,205]. For example, disruption of OGA activity mediates hyper *O*-GlcNAcylation, leading to muscle atrophy [58]. The possible underlying mechanisms of *O*-GlcNAcylation with regard to skeletal muscle atrophy are the following pathways: (1) as we previously discussed, *O*-GlcNAcylation seems to play a negative regulatory role on myogenesis as well as differentiation; (2) as we previously discussed, the differential regulation of *O*-GlcNAcylation on metabolism, signal transduction and stress response; (3) the regulation of *O*-GlcNAcylation on skeletal muscle contraction and structural properties such as calcium affinity, which is the most discussed topic at present. For details, please read to reference [56]; (4) the regulation of *O*-GlcNAcylation on synthesis and decomposition such as preventing proteasome degradation and mediating autophagy process and maintaining proteostasis [206], and this is what we need to discuss below. Skeletal muscle constantly finely regulates the movement and posture of the body, especially in daily exercise and physical activity, which produce a large amount of ROS to disrupt the normal physiological functions [207,208]. Therefore, skeletal muscle must not only efficiently recycle damaged or aged organelles and accumulated protein aggregates, but also efficiently decompose proteins to meet the energy requirements [209]. Autophagy, as a nutritional mechanism, is essential for removing aging and damaged cellular components, decomposing unallocated nutrient reserves and remodeling cellular structure [210]. The critical role of proper autophagic flux for skeletal muscle function and metabolism cannot be overstated [211]. Indeed, autophagy has been considered as an emerging metabolic regulatory pathway in skeletal muscle, and its role in skeletal muscle metabolism and atrophic diseases has been extensively studied [212,213]. In skeletal muscle autophagy signal transduction, mTOR, AMPK, AKT and FoxO3, as important central trophic effectors, regulate autophagy process according to nutritional status, and their interaction with OGT is inseparable [214,215,216,217]. Given the critical role of *O*-GlcNAcylation in these processes, *O*-GlcNAcylation may affect autophagy flux and proteostasis to regulate skeletal muscle mass and proteolysis [206]. For example, *O*-GlcNAcylation regulates autophagy flux and proteasome activity in the face of protein toxicity challenges [218]. A similar role in huntingtin toxicity was observed [219]. It should be noted that *O*-GlcNAcylation affects proteasome function and activity as well as assembly and changes cellular protein homeostasis [220]. *O*-GlcNAcylation of Rpt2 inhibits its activity to shut down the proteasome and cause protein accumulation [221,222]. *O*-GlcNAcylation of ryanodine receptor 1 increased ubiquitination and proteasomal degradation, thus triggering the cleavage of desmin filaments and the destruction of myofibrils [223]. Meanwhile, Caenorhabditis elegans bearing null mutations of *ogt-1* or *oga-1* increases autophagy under food deprivation-induced starvation [224,225]. Genetic ablation of OGT in mouse livers also affects autophagic flux under similar deprivation [25,216]. For example, the reduction of OGT in Drosophila was associated with the increase of autophagy-related gene (ATG) product and autophagy lysosomes [226]. OGT and *O*-GlcNAcylation in Caenorhabditis elegans affect the fusion of autophagosomes and lysosomes [227]. The *O*-GlcNAcylation level of synaptosome-associated protein 29 is also affected by nutritional availability, which eliminates its involvement in soluble *N*-ethylmaleimide-sensitive factor attachment protein receptor complex formation and thus disrupts the fusion between autophagosomes and endosomes/lysosomes [26,228]. Similarly, *O*-GlcNAcylation of Golgi reassembly stacking protein 55 acts as a tether to promote autophagosome maturation [229]. In addition, *O*-GlcNAcylation also participate in autophagy regulation by modifying other pivotal autophagy regulators such as beclin1, Bcl-2 and UNC-51-like kinase 1 (ULK1) [230]. See Figure 4 for more details.

## 5. Conclusions and Perspectives

In the last four decades, *O*-GlcNAcylation in skeletal muscle physiology has received increasing attention, but these understandings about its complex regulation and molecular mechanisms are still inadequate and initial. *O*-GlcNAcylation is a potential essential regulator for skeletal muscle glucose homeostasis and insulin sensitivity and mitochondrial biogenesis. Global *O*-GlcNAcylation patterns in skeletal muscle vary with rest, exertion or exercise and fiber type and thus differentially regulate a series of the pivotal signaling pathways and cellular stress responses as well as skeletal muscle plasticity and adaptation. A deeper understanding of these precise mechanisms by which *O*-GlcNAcylation functions is well worthwhile and a future hotspot. This future direction may lead to new valuable insights into skeletal muscle metabolism and exercise. *O*-GlcNAcylation is considered as a potential emerging mechanism to regulate contractile and structural properties of skeletal muscle. Future relevant studies should be considered in identifying skeletal muscle *O*-GlcNAcylated proteins critical for contractile activity and sarcomere cytoskeleton and their precise sites, while further focusing on their interactions and the dynamic crosstalk of *O*-GlcNAcylation with phosphorylation and other PTMs. The balance of phosphorylation and *O*-GlcNAcylation on myosin light chain 2 is a classic excellent example. *O*-GlcNAcylation is involved in skeletal muscle pathology in some related diseases such as atrophy, neuromuscular disease and insulin resistance, and it remains to be discussed as the cause or consequence of skeletal muscle impairments. *O*-GlcNAcylation has been recognized for mediating proteostasis in skeletal muscle through its benefits in autophagy, apoptosis and proteasomes. It deserves constant attention and the regulation of *O*-GlcNAcylation on myogenesis, differentiation and signaling pathways that maintain synthesis/degradation balance in skeletal muscle should not be neglected. Over time, further investigation of the pathological role of *O*-GlcNAcylation in skeletal muscle will provide the multiple novel therapeutic targets and the basis for developing new strategies to combat certain skeletal muscle diseases or metabolic disorders. There is a long way to go, although the future is bright and ambitious.
